# Editorial: Going the Distance: Enabling 3D Cell Culture Systems for Biomedical Research and Drug Treatment

**DOI:** 10.3389/fmolb.2021.685095

**Published:** 2021-05-10

**Authors:** Yong Teng, William C. Cho

**Affiliations:** ^1^Department of Oral Biology and Diagnostic Sciences, Dental College of Georgia, Augusta University, Augusta, GA, United States; ^2^Department of Biochemistry and Molecular Biology, Georgia Cancer Center, Medical College of Georgia, Augusta University, Augusta, GA, United States; ^3^Department of Medical Laboratory, Imaging and Radiologic Sciences, College of Allied Health, Augusta University, Augusta, GA, United States; ^4^Department of Clinical Oncology, Queen Elizabeth Hospital, Kowloon, China

**Keywords:** 3D cell culture systems, biomedical research, drug discovery, preclinical or clinical applications, translational science

Three-dimensional (3D) cell culture techniques are beginning to draw great attention as they allow different types of cells to be replicated *in vitro* while providing an accurate representation of cell growth *in vivo*. Given the remarkable potential of 3D cell culture to bridge the gap between 2D culture and native tissue, its increasing implementation in tissue engineering, disease modeling, and drug discovery is especially promising and suggestive that we are on track to obtaining more physiologically relevant information for both basic and translational sciences.

Owed to this advance, our Research Topic has garnered contributors that describe the current methodologies, applications, challenges facing 3D cell culture, practical insight into improving 3D techniques as well as advance 3D cell culture systems for biomedical research and drug treatment. After a rigorous peer-review process, eight articles have been accepted, which consist of one comprehensive review, one perspective, and six original research articles. Notably, these articles were contributed by academic institutes and biotechnology industries engaged in 3D cell culture work, demonstrating the great interest and applications in this hot area.

Our team has performed pioneer studies to take 3D cell culture research to a new level using a scaffold-based 3D cell culture system. We systemically reviewed the past and present 3D cell culture systems and discussed the challenges and prospects regarding their techniques and applications (Jensen et al.). Through this review, we urge other researchers to start transitioning from 2D to 3D cell cultures in order to open new avenues and shift the current research and clinical practice paradigm. It's worth mentioning that within 12 months of its publication, this article has received more views than 94% of all Frontiers articles.

Most available techniques for cellular rheology were developed for 2D systems, which has significantly hampered studies requiring proper cellular architecture. Chhetri et al., therefore, placed different cell culture models in perspective to illustrate how cellular mechanotransduction to the cell nucleus may be studied beyond standard 2D culture. They further highlighted that 3D cell culture platforms integrating different physical characteristics, as well as physical stress measurement methods, are needed to best render the phenotypic heterogeneity of cancers.

To solve the opposing requirements of hemoglobin-based oxygen delivery and cell function vs. experimental complexity and routine culture workflow, Shoemaker et al. presented a novel solution by developing a throughput-scalable organ-on-a-chip system comprised of a Perfused Organ Panel and PerfusionPal plates. They also introduced perfusion to circumvent the loss of cell signaling and drug metabolites in the otherwise one-way flow of perfusate. To address the challenging problem of early stages of cellular migration from multicellular tumor spheroids (MCTS) and cross-talk between spheroids, Shabalina et al. measured cell migration from MCTS derived from the NSCLC A549 cells cultured on different substrates, including collagen gel or plastic. This original research suggests patterns of circulating tumor cell migration can be predicted based on *ex vivo* analysis of patient biopsy in a 3D culture system. Microscopic imaging of tissue organoids and spheroids is challenging due to the light scattering nature of their 3D architecture. Recently, Edwards et al. demonstrated the advantages of combining expansion microscopy and immunolabeling of tumor spheroids and organoids. This group signified that the improved signal-to-background ratio of expanded samples greatly improved the subsequent methods for image segmentation and analysis. Furthermore, to offer a more efficient and cost-effective platform toward the discovery of novel treatment options for chondrosarcoma patients, Palubeckaite et al. developed a chondrosarcoma alginate spheroid model that was more representative of chondrosarcoma *in vivo* than the monolayer model for therapy testing.

Both cardiomyocytes (CM) and cardiac fibroblasts (CF) play essential roles in cardiac development, function, and remodeling; however, their properties of 3D co-cultures remain unclear. In this regard, Beauchamp et al. investigated characteristics of 3D single- and co-cultured hiPSC-CMs and CFs. By comparison with the standard 2D culture, they demonstrated that 3D culturing hiPSC-CMs and CFs could provide a new approach to mimic the natural organization of the heart more closely, especially by avoiding CF activation and myofibroblast transformation. ^64^CuCl_2_ is a promising theranostic agent for prostate cancer due to its potential to induce significant damage in cancer cells with minimal side effects in healthy tissues. To gain new insights into the cellular consequences of ^64^CuCl_2_ exposure in advanced culture systems, Pinto et al. assessed its effects on human prostate cancer multicellular tumor spheroids. Their results added weight toward understanding the mode of action of copper-based radiopharmaceuticals and their potential as a novel theragnostic compounds for prostate cancer.

Despite the fact that 3D cell culture is still in its infancy stage, it has opened the door to more faithfully recapitulating the complex aspects of human physiology, pathology, and drug response *in vitro*. Thereby, it is time to apply this technology to gain a better understanding and treatment of human diseases and the underlying biology.

## Author Contributions

YT drafted the editorial, which was input, revised, edited, and accepted by WC. All authors contributed to the article and approved the submitted version.

## Conflict of Interest

The authors declare that the research was conducted in the absence of any commercial or financial relationships that could be construed as a potential conflict of interest.

